# Effects of *PLIN1* Gene Knockout on the Proliferation, Apoptosis, Differentiation and Lipolysis of Chicken Preadipocytes

**DOI:** 10.3390/ani13010092

**Published:** 2022-12-26

**Authors:** Guiying Zhai, Yongjia Pang, Yichong Zou, Xinyu Wang, Jie Liu, Qi Zhang, Zhiping Cao, Ning Wang, Hui Li, Yuxiang Wang

**Affiliations:** 1 Key Laboratory of Chicken Genetics and Breeding, Ministry of Agriculture and Rural Affairs, Harbin 150030, China; 2 Key Laboratory of Animal Genetics, Breeding and Reproduction, Education Department of Heilongjiang Province, Harbin 150030, China; 3 College of Animal Science and Technology, Northeast Agricultural University, Harbin 150030, China

**Keywords:** lipocyte, lipid droplets, perilipin 1, function, CRISPR/Cas9

## Abstract

**Simple Summary:**

Perilipin 1 (PLIN1) is one of the most abundant lipid droplet-related proteins on the surface of adipocytes. In order to reveal the role of PLIN1 in the growth and development of poultry adipocytes, the CRISPR/Cas9 system was used to effectively mediate the knockout of the *PLIN1* gene. The results showed that PLIN1 deletion increased the proliferation, decreased, apoptosis and differentiation, promoted basal lipolysis and inhibited hormone-stimulated lipolysis of chicken preadipocytes. The results of this study are conducive to revealing the molecular mechanism of chicken PLIN1 regulating lipid metabolism, and to deeply understand the genetic mechanism of chicken adipose tissue growth and development.

**Abstract:**

Perilipin 1 (PLIN1) is one of the most abundant lipid droplet-related proteins on the surface of adipocytes. Our previous results showed that PLIN1 plays an important role in chicken lipid metabolism. To further reveal the role of PLIN1 in the growth and development of adipocytes, a chicken preadipocyte line with a *PLIN1* gene knockout was established by the CRISPR/Cas9 gene editing technique, and the effects of the *PLIN1* gene on the proliferation, apoptosis, differentiation and lipolysis of chicken preadipocytes were detected. The results showed that the CRISPR/Cas9 system effectively mediated knockout of the *PLIN1* gene. After the deletion of PLIN1, the differentiation ability and early apoptotic activity of chicken preadipocytes decreased, and their proliferation ability increased. Moreover, knockout of PLIN1 promoted chicken preadipocyte lipolysis under basal conditions and inhibited chicken preadipocyte lipolysis under hormone stimulation. Taken together, our results inferred that PLIN1 plays a regulatory role in the process of proliferation, apoptosis, differentiation and lipolysis of chicken preadipocytes.

## 1. Introduction

Lipid droplets (LDs) are ubiquitous cellular organelles that serve as the primary storage depot for energy and lipids in eukaryotic cells. In mammals, lipid droplets play an important role in cellular lipid homeostasis, primarily by storing either cholesterol esters to be used for membrane and steroid hormone synthesis or triacylglycerols that serve as a source of energy substrates or precursors for signalling lipids or membrane phospholipid synthesis. The structures of LDs comprise a neutral lipid core surrounded by a monolayer of phospholipids and specialized LD-associated surface proteins [1]. These proteins contribute to the biogenesis, maturation and stabilization of lipid droplets [2]. As the most abundant protein surrounding the lipid droplet in adipocytes, perilipin 1 (PLIN1) plays a crucial role in lipid homeostasis by regulating the access of lipases to neutral lipids in LDs. Depending on the energy state of the organism, PLIN1 either limits lipase access to stored triglycerides (in the fed state) or facilitates hormonally stimulated lipolysis (in the fasted state) [3]. Several in vivo and in vitro studies, including studies in perilipin-null mice, have confirmed that reduced levels or the absence of PLIN1 results in increased basal lipolysis. Mice with a systemic deletion of PLIN1 showed activated basal lipolysis and attenuated stimulated lipolysis [4].

Similar to the findings in mammals, PLIN1 was also shown to have a significant association with lipid metabolism in poultry. Research by our group showed that the chicken *PLIN1* gene is highly expressed in adipose tissue and gradually increases with the differentiation of preadipocytes. Meanwhile, overexpression of PLIN1 promotes lipid accumulation in chicken preadipocytes [5]. However, the associated effect of chicken PLIN1 on the growth and development of pre-adipocytes has not been fully characterized. Therefore, the present study aimed to investigate the role of PLIN1 in the proliferation, apoptosis, differentiation and lipolysis of chicken preadipocytes.

## 2. Materials and Methods

### 2.1. Cell Cultures and Differentiation

We established an immortalized chicken preadipocytes (ICP2) cell line by infecting primary chicken preadipocytes with recombinant retroviruses expressing chicken telomase reverse transcriptase and telomerase RNA [6]. The cells were cultured in Dulbecco’s modified Eagle’s medium/Nutrient Mixture F-12 (Gibco, Grand Island, NY, USA) containing with 10% fetal bovine serum (FBS) (BioInd, Kibbutz Beit Haemek, Israel) and incubated at 37 °C with 5% CO_2_.

When the ICP2 cells reached 60% confluence, the medium for inducing differentiation was used, which contained DMEM/F12 supplemented with 10% FBS and 260 μM sodium oleate (Sigma-Aldrich, St. Louis, MO, USA). The differentiation medium was changed every 24 h.

### 2.2. Establishment of a Chicken Preadipocyte Line with PLIN1 Knockout

With the CRISPR RGEN tool (http://www.genome.net/about/, accessed on 1 November 2018), eight highly specific gRNA spacers were designed to target exon 2, exon 4, exon 5 and exon 6 of the chicken *PLIN1* gene (GenBank accession number: NM_001127439.1). The oligonucleotide sequences of the gRNA (PAM sequence is underlined) are shown in Table 1.

The Poultry Cas9/gRNA Construction Kit (Beijing Weishang Lide Biotechnology Co., Ltd., Beijing, China) was used to construct the Cas9/gRNA vector according to the manufacturer’s instructions. The synthetic oligo dimers were inserted into the Cas9/gRNA vector and identified by transformation, coating, and sequencing.

When ICP2 cells reached 60% confluency, the Cas9/gRNA plasmids were transfected into the cell medium. After the cellular genomic DNA was extracted, the targeting efficiency of the corresponding gRNAs was estimated by comparing the proportion of DNA fragments digested with T7 endonuclease I (Vazyme, Nanjing, China). Then, the gRNA with the highest targeting efficiency was transfected into ICP2 cells, and positive cells were sorted by flow cytometry with green fluorescent protein (GFP) as a selection marker and cloned, propagated, and subcultured. After genomic DNA was extracted from single-cell clones, PCR analysis was performed to identify monoclones containing the target sites. The primer sequences used for PCR are shown in Table 2. The PCR product was sequenced, and the mutation pattern was analysed. Finally, the knockout effect was further confirmed by identification of the PLIN1 expression level in the filtered monoclonal cells by RT-qPCR and Western blotting.

### 2.3. Cell Proliferation Assay

Cell proliferation was examined using the Cell Counting Kit-8 (CCK-8) assay (Dojindo, Kyushu, Japan) and the Cell-Light EdU Apollo 567 In Vitro Kit (Ribobio, Guangzhou, China). For the CCK-8 assay, ICP2 cells were seeded on a 96-well plate at a density of 3.5 × 10^4^ cells/well. After 24 h, 10 μL of CCK-8 reagent was added to each well, and the plates were incubated at 37 °C for 1 h. The absorbance of each sample at a wavelength of 450 nm was detected using a microplate reader (Molecular Devices, San Jose, CA, USA). For EdU detection, 100 μL of 50 μM EdU medium was added to each well of the ICP2 cells, and the plates were incubated for 2 h to label the cells with EdU. Then, 50 μL of cell fixative (4% paraformaldehyde in PBS) was added to the cells, and the plates were incubated for 2 h to fix the cells. Afterwards, 100 μL of 1× Apollo staining reaction solution and 1× Hoechst 33342 reaction solution was added to each well, and the plates were incubated for 30 min in the dark at room temperature to stain the DNA of the cells with EdU. Finally, stained cell images were captured and analysed with a fluorescence microscope.

### 2.4. Cell Cycle Assay

The cell cycle was assessed using a Cell Cycle Testing Kit (Multisciences, Hangzhou, China). When the density reached 5 × 10^6^, ICP2 cells were collected and centrifuged at 800 rpm for 5 min. After being washed once with cold PBS, the cells were resuspended in 500 μL of propidium iodide staining solution and incubated at room temperature for 30 min in the dark. Then, the cell suspension was used for flow cytometry detection (Becton, Dickinson and Company, Franklin Lake, NJ, USA).

### 2.5. Cell Apoptosis Assay

Cell apoptosis was assessed by an annexin V-FITC/propidium iodide (PI) staining assay (Multisciences, Hangzhou, China). In brief, after being harvested by trypsinization, the ICP2 cells were resuspended in PBS and counted. Then, cells at a density of 5 × 10^4^ were incubated for 15 min in the dark at room temperature in the presence of annexin V-FITC (200 μL) and PI (10 μL). Afterwards, the cells were detected and analysed by flow cytometry (Becton, Dickinson and Company, Franklin Lake, NJ, USA).

### 2.6. Oil Red O Staining and Extraction Assay

The differentiated ICP2 cells were washed twice with PBS, fixed with 4% paraformaldehyde for 30 min at 4 °C, and rinsed 2 times with PBS and distilled water. The cells were stained with oil red O working solution (0.6% Oil Red O in isopropanol: water, 3:2) at room temperature for 15 min, then washed immediately with ddH_2_O. The cells were observed and analysed under a microscope (Leica, Wetzlar, Germany).

Lipid accumulation was measured by the oil red O extraction assay. After removing the staining solution, oil red O was extracted from the cells with 100% (*v*/*v*) isopropanol alcohol, and the absorbance at a wavelength of 510 nm was measured with a spectrophotometer (Biochrom, Cambridge, MA, USA).

### 2.7. RNA Extraction and RT-qPCR

Total RNA of ICP2 cells and PLIN-KO cells was extracted using a TRIzol reagent kit (Invitrogen, Grand Island, NY, USA) following the manufacturer’s protocol. cDNA generation was reverse-transcribed following the protocols of the ImProm IITM Reverse Transcription System (Takara, Da-lian, China). qPCR was conducted using a FastStart Universal SYBR Green Master kit (Roche Life Science, Indianapolis, IN, USA) on the 7500 real-time PCR system (Applied Biosystems, Foster City, CA, USA). Thermocycler settings were as follows: 95 °C for 10 min, 40 cycles at 95 °C for 15 s and 60 °C for 1 min. The relative expression level of target genes was calculated using the 2^−ΔΔCt^ method [7,8]. TATA-box binding protein (TBP) was used as the reference gene for normalization purposes. The sequences of the primers used for qPCR are shown in Table 3.

### 2.8. Western Blotting

For Western blot analysis, cell lysates mixed with 6× loading buffer (Beyotime, Shanghai, China) were denatured at 100 °C for 5 min and separated on a 10% SDS-PAGE gel(Bio-Rad, Hercules, CA, USA). The proteins were transferred to a nitrocellulose (NC) membrane (Millipore, Boston, MA, USA). Then, the membrane was blocked for 2 h and incubated overnight with the appropriate primary antibody (Perilipin-1, Cell Signaling Technology, Danvers, MA, USA) at 4 °C. The next day, the membrane was incubated with an HRP-conjugated secondary antibody (ZSGB-Bio, Beijing, China) for 1 h at room temperature. Specific protein bands were visualized using the ECL Plus Detection Kit (Beyotime, Shanghai, China) with a chemiluminescence system (Sagecreation, Beijing, China) and ImageQuant LAS 500 system (GE, Piscataway, NJ, USA). 

### 2.9. Triglyceride (TG) Assay

Following the protocols of the triglyceride (TG) detection kit of Nanjing Jiancheng Bioengineering Institute (Nanjing, China), chicken preadipocytes induced by oleic acid for 24 hours were collected. Then, 0.2–0.3 mL of lysis buffer (TritionX-100, 1–2%) was added to the cell pellet, and it was incubated for 30–40 min. Distilled water, calibrator and 2.5 μL sample were added to blank wells, standard wells, and sample wells on a 96-well plate, then 250 μL of working solution was added to each well. The OD value of each well was measured by an enzyme labelling instrument at 510 nm. The triglyceride content was calculated within the linear range of the standard curve and corrected by the concentration of each mg protein in the homogenate.

$$\mathrm{Triglyceride}\;\mathrm{content}:\;(\mathrm{mmol}/\mathrm{gprot})=\frac{A\;\mathrm{sample}\;-\;A\;\mathrm{blank}}{\;A\;\mathrm{standard}\;-\;A\;\mathrm{blank}}\;\times \;C\;\mathrm{standard}/\mathrm{Cpr}$$

C standard: standard concentration, 2.26 mmol/L

Cpr: homogenate protein concentration of the sample to be tested, gprot/L

### 2.10. Assay of HSL and ATGL Activity

The protocols of the chicken fat triglyceride ELISA detection kit and chicken hormone sensitive lipase ELISA detection kit from Zhenge Biological Company (Shanghai, China) were followed. Preadipocytes induced by oleic acid for 24 hours were removed, the supernatant of the culture medium was collected, and the particles and polymers were removed by centrifugation at 3000 rpm for 10 min. In a 96-well plate, 50 μL of different concentrations of standards were added to the standard wells, and 10 μL of samples and 40 μL of sample diluent were added to the sample wells. Then, 100 μL of horseradish peroxidase (HRP)-labelled detection antibody was added to each well, and the reaction wells were sealed with sealing film and incubated in an incubator for 60 min. After washing with detergent, 50 μL of substrate A and 50 μL of substrate B were added to each well, and the plates were incubated at 37 °C for 15 min in the dark. Then, 50 μL of Terminator solution was added to each well, and the OD of each well was measured at a wavelength of 450 nm for 15 minutes. A standard curve was drawn, and the concentration of each sample was calculated.

### 2.11. Statistical Analysis

The experimental data in this study were analysed using GraphPad Prism (5.0, Harvey Motulsky, Graphpad, San Diego, CA, USA) and JMP software (JMP Pro 16, John Sall, SAS, Rochester, NY, USA) and are presented as the means ± standard deviations (SD). The double-tailed Student’s t test was used to compare results between two groups. Differences were considered statistically significant at *p* < 0.05. In each experiment, three biological replicates were performed in each group, and all experiments were repeated at least three times independently.

## 3. Results

### 3.1. Establishment of a Chicken PLIN1 Knockout Preadipocyte Cell Line

**Figure 1:**
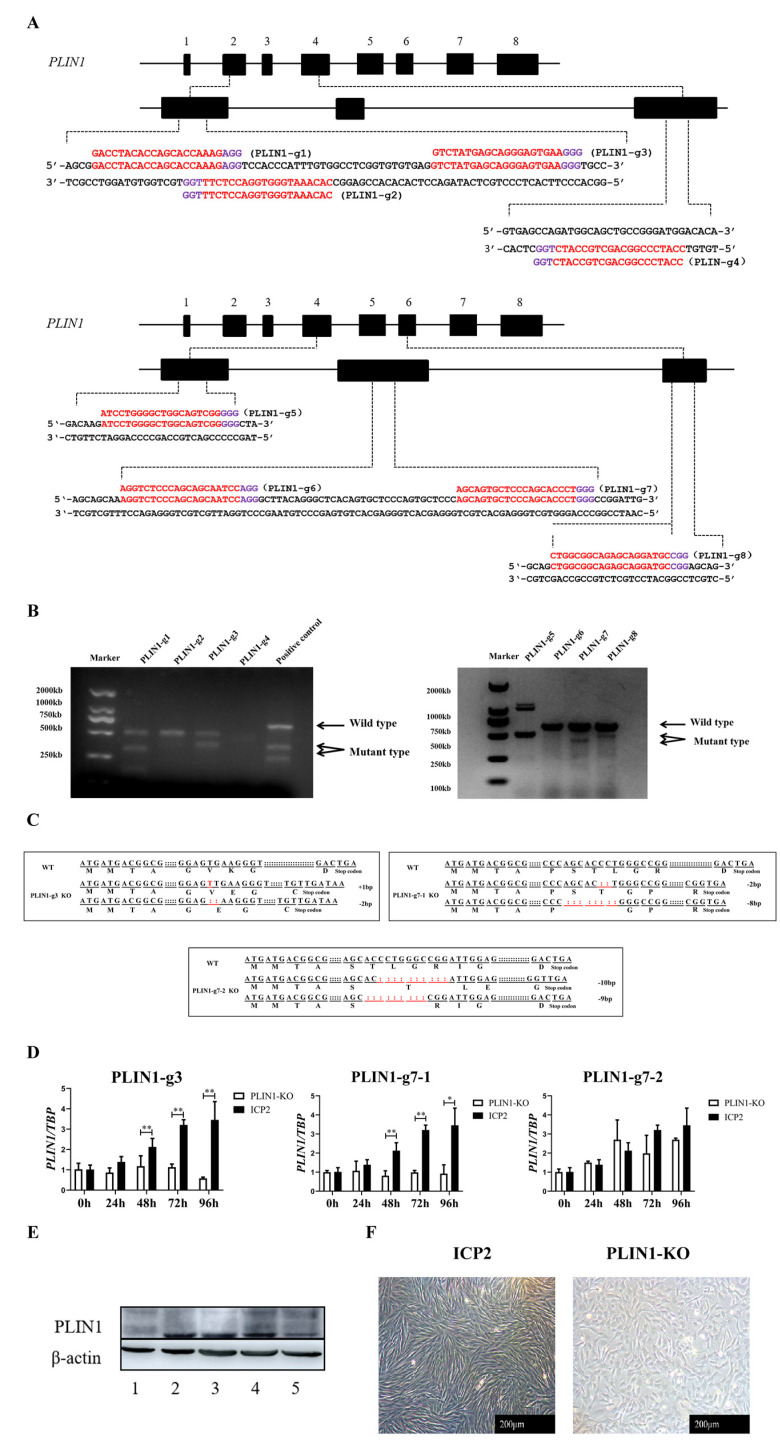
Establishment of a chicken PLIN1 knockout preadipocyte line. (**A**): gRNA target sites in the chicken *PLIN1* gene. Exons are shown as black boxes. The gRNA-targeting sequence is labelled in red, and the protospacer-adjacent motif (PAM) sequence is labelled in purple. (**B**): Cleavage activity assay of gRNA. (**C**): Sequencing results and gene editing type analysis. Red indicates inserted or missing bases. (**D**): Detection of the mRNA expression level of the *PLIN1* gene in ICP2 and PLIN1-KO cell lines. (**E**): Detection of the protein expression level of PLIN1 in ICP2 and PLIN1-KO cell lines. Lane 1: ICP2 cells transfected with PLIN1-g7-1; Lane 2: ICP2 cells; Lane 3: ICP2 cells transfected with PLIN1 overexpression plasmids; Lane 4: ICP2 cells transfected with PLIN1-g7-2; Lane 5: ICP2 cells transfected with PLIN1-g3. (**F**): Effect of PLIN1 knockout on the morphology of chicken preadipocytes. All data are representative of 3 independent experiments and are shown as the means ± SDs (* *p* < 0.05, ** *p* < 0.01).

To obtain the greatest chicken PLIN1 knockout efficiency, eight gRNAs encoding portions of the chicken *PLIN1* gene were designed: PLIN1-g1, PLIN1-g2, and PLIN1-g3 in exon 2; PLIN1-g4 and PLIN1-g5 in exon 4; PLIN1-g6 and PLIN1-g7 in exon 5; and PLIN1-g8 in exon 6. The primer sequences and target sites of these gRNAs are shown in Figure 1A. Then, eight Cas9 RNPs were constructed by mixing these gRNAs with the Cas9 protein and were transfected into ICP2 cells. The results of genome cleavage activity analysis showed that PLIN1-g1-, PLIN1-g3-, PLIN1-g7- and PLIN1-g8-transfected cells had higher cleavage activity (Figure 1B). Therefore, these four gRNA-transfected cells were selected for monoclonal screening. A total of 24 monoclonal cells were identified in this study. Sanger sequencing results showed that three monoclonal cells had successful homozygous knockout of the *PLIN1* gene, resulting in amino acid frameshift mutations that prematurely terminated protein translation (Figure 1C). Then, the results of RT-qPCR showed that the mRNA expression level of the chicken *PLIN1* gene in homozygous knockout monoclonal cells transfected with PLIN1-g3 and PLIN1-g7-1 was significantly decreased compared with that in ICP2 cells at 48 h, 72 h, and 96 h of differentiation (*p* < 0.01, Figure 1D). Meanwhile, Western blotting confirmed that the expression of chicken PLIN1 protein in the monoclonal cells transfected with PLIN1-g7-1 was significantly lower than that in ICP2 cells (Figure 1E), which indicated that the chicken PLIN1 protein in these monoclonal cells had been knocked out. After large-scale culture, these monoclonal cells were named PLIN1-KO cells, which defined them as chicken preadipocyte lines with PLIN1 protein knockout.

In addition, comparison of cell morphology between PLIN1-KO cells and ICP2 cells showed that the lack of PLIN1 protein changed the morphology of the chicken preadipocytes from fusiform to oval (Figure 1F).

### 3.2. Knockout of PLIN1 Promotes Proliferation of Chicken Preadipocytes

**Figure 2:**
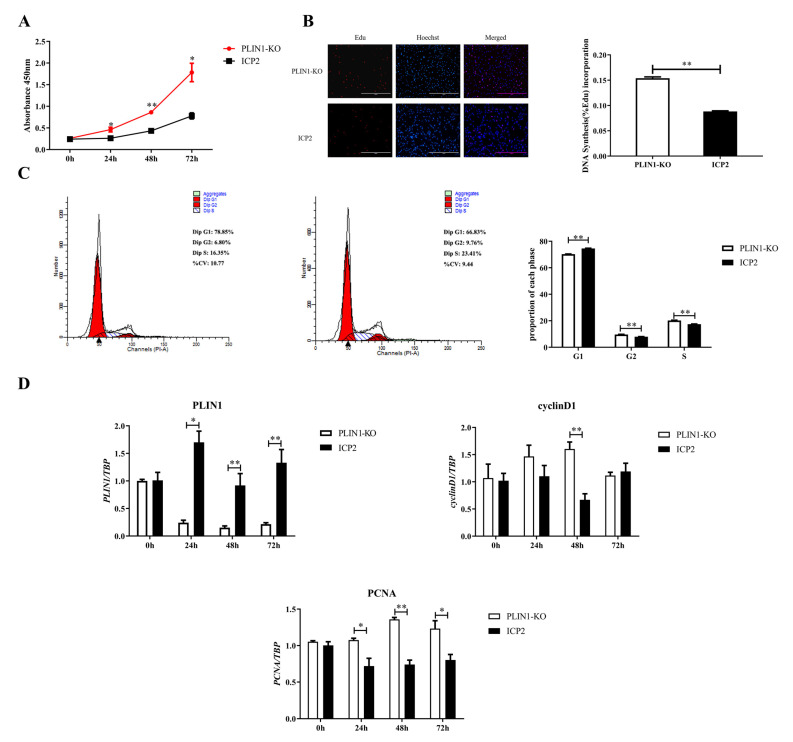
Knockout of PLIN1 promotes the proliferation of chicken preadipocytes. Twelve hours after inoculation, cell proliferation was recorded as 0 h. (**A**): Effect of PLIN1 knockout on chicken preadipocyte proliferation rate. (**B**): Effect of PLIN1 knockout on chicken preadipocyte DNA synthesis. (**C**): Effect of PLIN1 knockout on the chicken preadipocyte cycle. (**D**): Effect of PLIN1 knockout on the mRNA expression of cell proliferation marker genes. All data are representative of three independent experiments and are shown as the means ± SDs (* *p* < 0.05, ** *p* < 0.01).

First, the effects of PLIN1 knockout on the proliferation rate of chicken preadipocytes were detected with a CCK-8 assay. The results showed that at 24, 48 and 72 h of cell proliferation, the proliferation rate of PLIN1-KO cells was significantly higher than that of ICP2 cells (*p* < 0.05, Figure 2A). Second, the EdU staining assay was applied to analyse the reason why knockout of PLIN1 affected the proliferation rate of chicken preadipocytes. The results showed that at 48 h of cell proliferation, the DNA synthesis rate of PLIN1-KO cells was significantly higher than that of ICP2 cells (*p* < 0.01, Figure 2B). Third, the PI staining method was used by flow cytometry to explore the role of PLIN1 deletion on the cell cycle of chicken preadipocytes. The results showed that compared with that in ICP2 cells, the proportion of PLIN1-KO cells in the G1 phase was significantly decreased (*p* < 0.01), and the proportion of cells in the S and G2 phases was significantly increased (*p* < 0.01, Figure 2C). The results indicate that knockout of PLIN1 promoted the progression of chicken preadipocytes from G1 to S phase. Fourth, to further confirm the effect of PLIN1 knockout on the proliferation of chicken preadipocytes, RT-qPCR was performed to compare differences in the mRNA expression levels of the cell proliferation marker genes, *cyclinD1* and *PCNA* between the two cell lines, and the results are shown in Figure 2D. The mRNA expression levels of the *cyclinD1* gene in PLIN1-KO cells were significantly higher than that in ICP2 cells at 48 h (*p* < 0.01), and the mRNA expression levels of the *PCNA* gene in PLIN1-KO cells was significantly higher than that in ICP2 cells at 24, 48 and 72 h (*p* < 0.05). As expected, at all detected time points, the mRNA expression levels of the *PLIN1* gene in PLIN1-KO cells were significantly lower than those in ICP2 cells (*p* < 0.05 or *p* < 0.01).

### 3.3. Knockout of PLIN1 Suppressed Apoptosis of Chicken Preadipocytes

**Figure 3:**
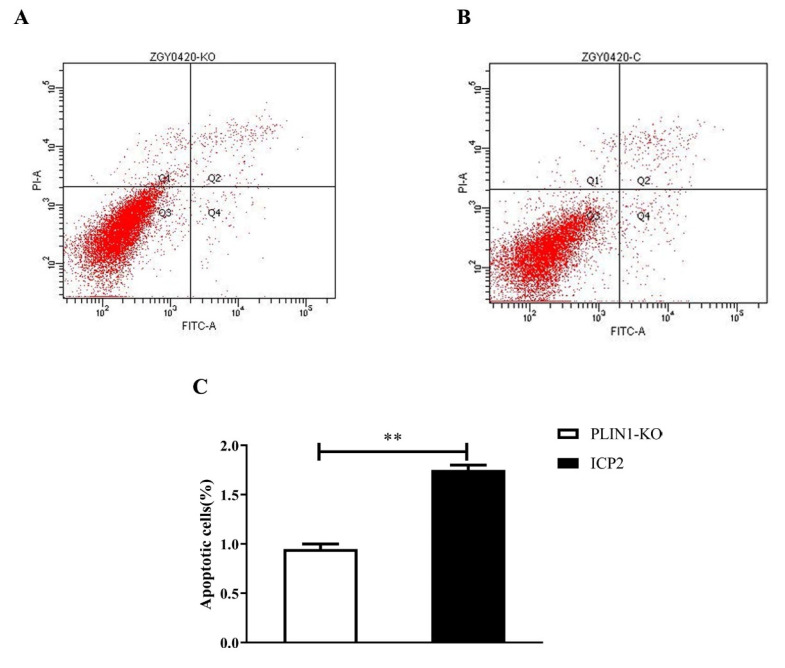
Knockout of PLIN1 suppressed apoptosis of chicken preadipocytes. (**A**): The proportion of apoptosis in the PLIN1-KO cell line. (**B**): The proportion of apoptosis in the ICP2 cell line. (**C**): Effect of PLIN1 knockout on chicken preadipocyte apoptosis. All data are representative of three independent experiments and are shown as the means ± SDs (** *p* < 0.01).

To detect the effect of PLIN1 knockout on chicken preadipocyte apoptosis, an annexin V-FITC staining assay was performed. The results showed that when the cells proliferated for 48 h, the proportion of apoptotic cells in the PLIN1-KO cells was extremely significantly reduced compared with that in the ICP2 cells (*p* < 0.0 l, Figure 3C), indicating that PLIN1 very significantly promotes the apoptosis of chicken preadipocytes.

### 3.4. Knockout of PLIN1 Inhibited the Differentiation of Chicken Preadipocytes

**Figure 4:**
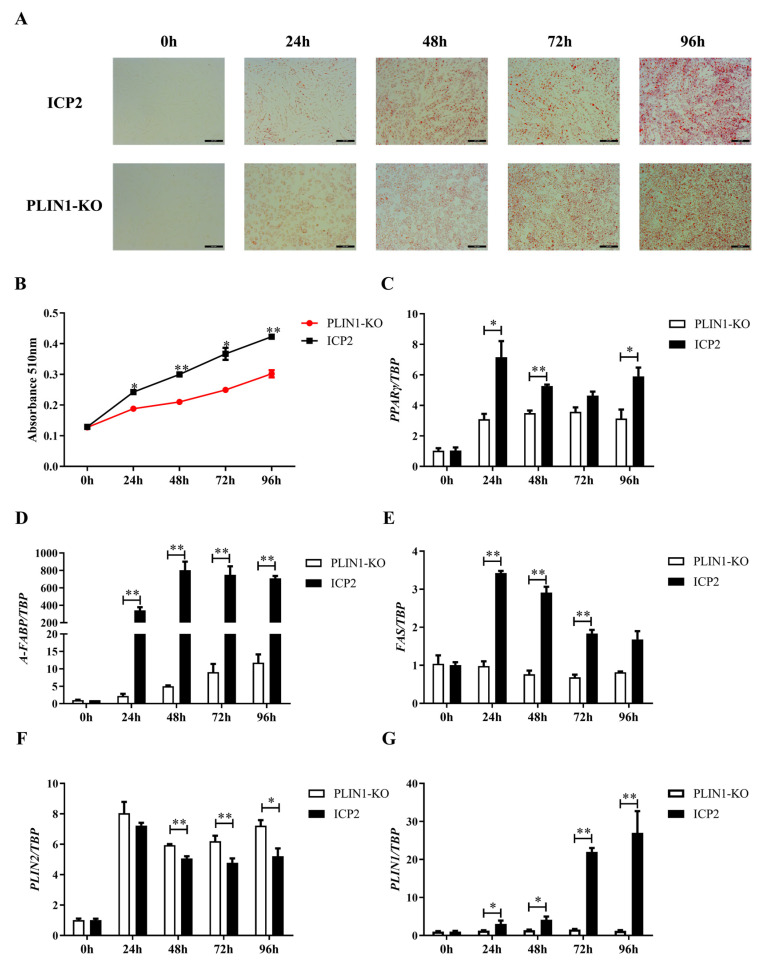
Knockout of PLIN1 inhibited the differentiation of chicken preadipocytes. (**A**,**B**): Comparison of LD accumulation during preadipocyte differentiation between ICP2 cells and PLIN1-KO cells. (**A**): Oil red O staining (200 μM). (**B**): Oil red O extraction. (**C**–**G**): Effect of *PLIN1* gene knockout on the expression of preadipocyte differentiation marker genes. (**C**): *PPARγ* gene; (**D**): *A-FABP* gene; (**E**): *FAS* gene; (**F**): *PLIN2* gene; (**G**): *PLIN1* gene. All data are representative of 3 independent experiments and are shown as the means ± SDs (* *p* < 0.05, ** *p* <0.01).

To investigate the effect of PLIN1 knockout on the differentiation of chicken preadipocytes, oil red O staining and extraction assays were carried out. As shown in Figure 4A, at different stages of differentiation, the intracellular lipid accumulation in PLIN1-KO cells was significantly less than that in ICP2 cells. The lipid content of PLIN1 knockout preadipocytes was significantly lower than that of wild-type preadipocytes at 24, 48, 72, and 96 h of cell differentiation (*p* < 0.05 or *p* < 0.01, Figure 4B). Consistent with these findings, RT-qPCR analysis of adipogenic marker gene expression showed that the mRNA expression level of the *PPARγ* gene in PLIN1-KO cells was significantly lower than that in ICP2 cells at 24, 48 and 96 h of cell differentiation (*p* < 0.05 or *p* < 0.01, Figure 4C), the mRNA expression level of the *A-FABP* gene in PLIN1-KO cells was significantly lower than that in ICP2 cells at 24, 48, 72 and 96 h of cell differentiation (*p* < 0.01, Figure 4D), the mRNA expression level of the *FAS* gene in PLIN1-KO cells was significantly lower than that in ICP2 cells at 24, 48 and 72 h of cell differentiation (*p* < 0.01, Figure 4E), and the mRNA expression level of the *PLIN2* gene in PLIN1-KO cells was significantly higher than that of ICP2 cells at 48, 72 and 96 h of cell differentiation (*p* < 0.05 or *p* < 0.01, Figure 4F). As expected, the mRNA expression level of the *PLIN1* gene in PLIN1-KO cells was significantly lower than that in ICP2 cells at different time points of cell differentiation (*p* < 0.05 or *p* < 0.01, Figure 4G). Collectively, these results suggest that PLIN1 promotes the differentiation of chicken preadipocytes.

### 3.5. Effect of PLIN1 Knockout on Lipolysis in Chicken Preadipocytes

**Figure 5:**
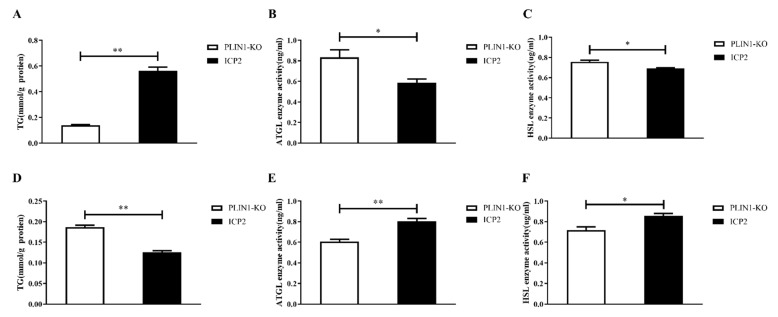
Effect of *PLIN1* gene knockout on TG content and HSL and ATGL activity in chicken preadipocytes under basal conditions and hormone stimulation. (**A**,**D**): Effect of *PLIN1* gene knockout on TG content in chicken preadipocytes. (**A**): basal condition; (**D**): hormone stimulation. (**B**,**E**): Effect of *PLIN1* gene knockout on ATGL activity in chicken preadipocytes. (**B**): basal condition; (**E**): hormone stimulation. (**C**,**F**): Effect of knockout of the *PLIN1* gene on the activity of HSL in chicken preadipocytes. (**C**): basal condition; (**F**): hormone stimulation. All data are representative of three independent experiments and are shown as the means ± SDs (* *p* < 0.05, ^**^ *p* < 0.01).

To explore the effect of PLIN1 knockout on the lipolysis of chicken preadipocytes, we compared the differences in TG content and HSL and ATGL activity in PLIN1-KO and ICP2 cells under basal and hormone-stimulated conditions. Under basal conditions, after differentiation for 24 h, the TG content in PLIN1-KO cells was significantly lower than that in ICP2 cells (*p* < 0.01, Figure 5A), and the ATGL and HSL activities in PLIN1-KO cells were significantly higher than those in ICP2 cells (*p* < 0.05 or *p* < 0.01, Figure 5B,C). Under isoproterenol stimulation for 6 h after differentiation for 24 h, the TG content in PLIN1-KO cells was significantly higher than that in ICP2 cells (*p* < 0.01, Figure 5D), and the ATGL and HSL activities in PLIN1-KO cells were significantly lower than those in ICP2 cells (*p* < 0.01, Figure 5E,F). These results provide evidence that PLIN1 inhibits the lipolysis of chicken adipocytes under basal conditions and promotes the lipolysis of chicken adipocytes under hormone stimulation.

## 4. Discussion

PLIN1 is the first lipid droplet coating protein found to be localized in lipid droplets and to regulate TAG storage and hydrolysis [9]. As the most abundant lipid droplet-related protein on the surface of lipid droplets, PLIN1 plays an important role in regulating lipid droplet formation and lipid metabolism. Our previous research on the tissue expression of the chicken *PLIN1* gene [10,11] and the effect of PLIN1 on the lipid accumulation of chicken preadipocytes [5], showed that chicken PLIN1 has similar functions to mammalian PLIN1. However, the role of PLIN1 in the proliferation, apoptosis and lipolysis of chicken preadipocytes remains unclear. To this end, we established chicken preadipocytes with PLIN1 protein knockout using CRISPR/Cas9 technology. The proliferative capacity of chicken preadipocytes increased, and the apoptosis and differentiation capacity decreased after the deletion of PLIN1. Moreover, knockout of PLIN1 promoted the lipolysis of chicken preadipocytes under basal conditions and inhibited the lipolysis of chicken preadipocytes under hormone stimulation.

Obtaining sgRNAs with high targeting efficiency is a critical step when using CRISPR/Cas9 technology to effectively knock out target genes [12]. In this study, we designed multiple sgRNAs for the chicken *PLIN1* gene. According to the bioinformatics prediction score [13], eight sgRNAs were selected, and the targeting efficiency of each sgRNA in vitro was tested. According to the grey value comparison result of the mutant band and the wild-type band, four sgRNAs with high targeting efficiency were identified: PLIN1-g1, PLIN1-g3, PLIN1-g7, and PLIN1-g8 (Figure 1B). Subsequently, we screened and obtained three homozygous knockout monoclonal cells. Although the mRNA expression level of the chicken *PLIN1* gene was significantly decreased in these monoclonal cells (Figure 1D), only PLIN1-g7-1 significantly downregulated the protein expression level of chicken PLIN1 (Figure 1E). Therefore, we propagated the cell line transfected with PLIN1-g7-1 and used it as a PLIN1 knockout chicken preadipocyte line for subsequent studies. Investigation of the mechanism of PLIN1 knockout by PLIN1-g7-1 showed that the target site of PLIN1-g3 did not fall in the common region of chicken PLIN1 protein isoforms (chicken PLIN1 has four protein isoforms, unpublished data); therefore, we failed to knock out all protein isoforms of PLIN1. For PLIN1-g7-2, we found that the gRNA caused the deletion of nine bases on a homologous chromosome, which only resulted in a partial deletion of amino acids from the PLIN1 protein and did not achieve true homozygous knockout; therefore, no significant change in the protein level of chicken PLIN1 was detected.

It was interesting that in the present study, we observed that knockout of PLIN1 transformed chicken preadipocytes from the original spindle shape to an oval shape (Figure 1F). These results showed that as a key lipid droplet coating protein, knockout of PLIN1 may cause the cells to lose their protection on lipid droplets, leading to changes in the morphology of chicken preadipocytes.

The proliferation of preadipocytes is tightly regulated. In addition to being influenced by a variety of hormones, some cytokines, such as transforming growth factor-β, tumour necrosis factor-α, macrophage-colony stimulating factor, angiotensin II, basic fibroblast growth factor, and bone morphogenetic protein, also have positive or negative regulatory effects on the proliferation of adipocytes [14,15,16,17,18,19,20,21,22,23]. Mammalian studies have shown that PLIN1 plays an important role in preadipocyte differentiation [24,25], but whether it has an effect on preadipocyte proliferation is unclear. To this end, this study examined the effect of PLIN1 on the cell viability and DNA synthesis activity of chicken preadipocytes, and the results of CCK-8 and EdU assays showed that knockout of PLIN1 promoted the proliferation of chicken preadipocytes (*p* < 0.01, Figure 2A,B). Studies have shown that changes in the cell cycle can regulate cell proliferation [26,27]. To explore the possible mechanism by which PLIN1 knockout promotes the proliferation of chicken preadipocytes, we first detected the cell cycle changes in Plin1-KO cells by flow cytometry. Knockout of PLIN1 promoted the progression of chicken preadipocytes from G1 phase to S phase (*p* < 0.01, Figure 2C). Subsequently, we detected changes in the mRNA expression levels of cell proliferation marker genes (*cyclinD1 and PCNA*) in PLIN1-KO cells. CyclinD1 is a member of the cyclin family and can accelerate the transition of the cell cycle from G1 phase to S phase [28,29]. Proliferating cell nuclear antigen (PCNA) can control the cell cycle through direct interaction with the cyclin/cdk complex, pushing the cell cycle from G1 to S phase. The results of this study showed that the mRNA expression levels of *cyclinD1* and *PCNA* genes in the cells of the PLIN1-KO group were significantly higher than those of the ICP2 cell group during cell proliferation (Figure 2D). Based on these findings, it can be concluded that promotion of chicken preadipocyte proliferation by knockout of PLIN1 occurs through promotion of the expression of genes related to cell proliferation, which further pushes the cell cycle.

Using annexin V-FITC staining, we found that knockout of the *PLIN1* gene had a very significant inhibitory effect on the apoptosis of chicken preadipocytes (*p* < 0.01, Figure 3A,B). PCNA inhibits apoptosis by interacting with pro-apoptotic factors (ING1b) and anti-apoptotic factors (P53, GADD 45, MyD 118, and CR6) [30]. In the present study, the mRNA expression level of *PCNA* was significantly upregulated in PLIN1-KO cells relative to ICP2 cells (*p* < 0.01, Figure 3C), further indicating that knockout of PLIN1 has an inhibitory effect on apoptosis in chicken preadipocytes.

Our current findings in chickens, and previous findings in mammals, suggest that overexpression of PLIN1 promotes lipid deposition [5,31]. In the present study, quantification of oil red O staining intensity showed that knockout of PLIN1 reduced lipid deposition in chicken preadipocytes during differentiation (*p* < 0.01, Figure 4A,B). Mammalian studies have shown that the growth of lipid droplets in adipocytes is mainly caused by an increase in the rate of fat synthesis, an increase in the rate of lipid droplet fusion, or a decrease in the rate of fat degradation [31,32]. Studies have shown that PLIN1 can interact with the CIDE-N domain of Fsp27, change the conformation of Fsp27, and promote the efficient fusion of lipid droplets in adipocytes, thereby promoting the growth of lipid droplets [33]. Therefore, on the one hand, the decrease in lipid deposition may be due to the deletion of PLIN1 causing lipid droplets to lose their protection and lipolases to gain more access to lipid droplets, promoting lipolysis. On the other hand, knockout of PLIN1 may attenuate the effect of Fsp27 in promoting lipid droplet fusion, resulting in slower lipid droplet growth and reduced lipid deposition. *PPARγ, A-FABP,* and *FAS* are important marker genes of adipocyte differentiation and maturation, and play an important role in the process of fat deposition [34,35,36]. The results of this study showed that compared with those in wild-type preadipocytes, the expression levels of the *PPARγ, A-FABP,* and *FAS* genes in the PLIN1 knockout group were significantly downregulated during cell differentiation (*p* < 0.01, Figure 4C–E). This is consistent with the results in mice: transcriptional levels of adipogenic genes, such as *C/EBPs*, *SREBP-1c*, *PPARγ*, *A-FABP*, *FAS*, *HSL,* and *ATGL,* were downregulated during preadipocyte differentiation in PLIN1^−/−^ mice [25]. The above findings suggest that knockout of the *PLIN1* gene attenuates the ability of chicken preadipocytes to differentiate into adipocytes. That is, PLIN1 promotes lipid deposition in chicken preadipocytes. Studies have shown that PLIN2 also plays an important role in regulating lipid metabolism [37,38,39]. When cells differentiate into mature adipocytes, PLIN2 disappears on the surface of lipid droplets or is completely replaced by PLIN1 [40,41], indicating that the two proteins have a certain similarity in function and can be compensatively expressed. The results of this study showed that the expression of the *PLIN2* gene had an upward trend in chicken preadipocytes with PLIN1 knockout, which was consistent with a study in cattle [42]. This indicates that knockout of PLIN1 can compensatively increase the expression of the *PLIN2* gene. This may occur because in order to maintain normal cell shape and the stability of lipid droplets, and inhibit the lipolysis of triglycerides, PLIN1-KO cells trigger the compensatory expression of other genes in the PLIN family.

Studies in mammals, such as mice, have shown that PLIN1 plays a dual regulatory role in adipocyte lipolysis [43]. Triglycerides (TGs) are an important component of lipids in the animal body, and are mainly stored in adipose tissue. The activation of adipocyte lipolysis requires three lipases: adipose triglyceride lipase (ATGL), hormone-sensitive lipase (HSL) and monoacylglycerol lipase (MAGL). Among them, ATGL, belonging to the patatin-like phospholipase domain protein family, is the most critical rate-limiting enzyme; it is essentially a lipid droplet protein and is highly expressed in adipose tissue [44]. It has been reported that PLIN1 promotes ATGL redistribution on lipid droplets, thereby enhancing ATGL-mediated lipolysis [45]. HSL is a multifunctional enzyme involved in fatty acid metabolism and is highly expressed in adipose tissue [46]. In adipocytes, PLIN1 is essential for functional lipolytic activation and is closely associated with HSL translocation and activation [47]. In this study, we found that compared with wild-type cells, under basal conditions, the content of triglycerides in the cells of the PLIN1-KO group was significantly decreased (Figure 5A, *p* < 0.01), and the activities of HSL and ATGL were significantly increased (Figure 5B,C, *p* < 0.01). In contrast, in the state of hormone stimulation, the triglyceride content was significantly increased (Figure 5D, *p* < 0.01), and the HSL and ATGL activities were significantly decreased (Figure 5E,F, *p* < 0.01). These results indicate that knockout of PLIN1 promotes the lipolysis of chicken preadipocytes under basal conditions and can inhibit the lipolysis of chicken preadipocytes in the hormone-stimulated state, which is consistent with the results of mammalian studies [48,49,50].

## 5. Conclusions

In this study, a chicken preadipocyte line with PLIN1 knockout was successfully established using CRISPR/Cas9 technology. At the same time, it was found that knocking out PLIN1 has a certain promotional effect on the proliferation of chicken preadipocytes, and has a certain inhibitory effect on the apoptosis and differentiation of chicken preadipocytes. Moreover, deletion of PLIN1 has a certain promoting effect on the lipolysis of chicken preadipocytes under basal conditions and has a certain inhibitory effect on the lipolysis of chicken preadipocytes under hormone stimulation. The results of this study are conducive to revealing the molecular mechanism of chicken PLIN1 regulating lipid metabolism, and to deeply understand the genetic mechanism of chicken adipose tissue growth and development, which lays a theoretical foundation for the ultimate control of excessive fat deposition in chickens. 

## Figures and Tables

**Table 1:** Oligonucleotide sequences used in the construction of PLIN1 gRNA.

Name	Oligonucleotide Sequence (5’-3’)
PLIN1-2e-g1	GACCTACACCAGCACCAAAGAGG
PLIN1-2e-g2	CACAAATGGGTGGACCTCTTTGG
PLIN1-2e-g3	GTCTATGAGCAGGGAGTGAAGGG
PLIN1-4e-g4	CCATCCCGGCAGCTGCCATCTGG
PLIN1-4e-g5	ATCCTGGGGCTGGCAGTCGGGGG
PLIN1-5e-g6	AGGTCTCCCAGCAGCAATCCAGG
PLIN1-5e-g7	AGCAGTGCTCCCAGCACCCTGGG
PLIN1-6e-g8	CTGGCGGCAGAGCAGGATGCCGG

**Table 2:** PCR detection primers.

Primer Name	Primer Sequence (5’-3’)
PLIN1-2e-F	TCCCCATGGTGGACCAAAAA
PLIN1-2e-R	GCAGGTACTGTCCCACTGTT
PLIN1-4e-F	TTCTGTATGGCTGGCTACCTC
PLIN1-4e-R	CTTCCTCCACCTCAGCAGAAC
PLIN1-5/6e-F	GAGAAAGATCCTGGTGCAGGG
PLIN1-5/6e-R	GAGAACATGGTGGGACTGAGA

**Table 3:** Primers used for qPCR.

Gene ^1^	Accession Number	Primer Sequence (5ʹ to 3ʹ)
*PPAR*γ	NM_001001460	F: GTGCAATCAAAATGGAGCC R: CTTACAACCTTCACATGCAT
*A-FABP*	NM_204290	F: ATGTGCGACCAGTTTGT R: TCACCATTGATGCTGATAG
*PLIN1*	NM_001127439	F: GGGGTGACTGGCGGTTGTA R: GCCGTAGAGGTTGGCGTAG
*FAS*	NM_205155	F: AAGGCGGAAGTCAACGG R: TTGATGGTGAGGAGTCG
*PLIN2*	NM_001031420.1	F:TCTTGGGAAGTCGTGTGGTG
		R: CACGTGCACGGAACTTTGAA
*TBP*	NM_205103	F:GCGTTTTGCTGCTGTTATTATGAG
		R:TCCTTGCTGCCAGTCTGGAC
*CyclinD1*	NM_205381	F:CTCGGAGCTACCTGCATGTTT
		R:GTTTACGGATGATCTGTTTGGTG
*PCNA*	NM_204170	F:GTGCTGGGACCTGGGTT
		R:CGTATCCGCATTGTCTTCTG

^1^ Gene name.

## Data Availability

The data presented in this study are available on request from the corresponding author.
